# Impact of a New Law to Reduce the Legal Blood Alcohol Concentration Limit– A Poisson Regression Analysis and Descriptive Approach

**Published:** 2017-03-31

**Authors:** Beatriz Nistal-Nuño

**Affiliations:** ^1^ Stanford University Medical Center, Emergency Medicine Department, Palo Alto, USA

**Keywords:** Poisson, Public health, Blood alcohol concentration, Fatalities, Injuries

## Abstract

**Background:** In Chile, a new law introduced in March 2012 lowered the blood alcohol concentration (BAC) limit for impaired drivers from 0.1% to 0.08% and the BAC limit for driving under the influence of alcohol from 0.05% to 0.03%, but its effectiveness remains uncertain. The goal of this investigation was to evaluate the effects of this enactment on road traffic injuries and fatalities in Chile.

**Study design:** A retrospective cohort study.

**Methods:** Data were analyzed using a descriptive and a Generalized Linear Models approach, type of Poisson regression, to analyze deaths and injuries in a series of additive Log-Linear Models accounting for the effects of law implementation, month influence, a linear time trend and population exposure. A review of national databases in Chile was conducted from 2003 to 2014 to evaluate the monthly rates of traffic fatalities and injuries associated to alcohol and in total.

**Results:** It was observed a decrease by 28.1 percent in the monthly rate of traffic fatalities related to alcohol as compared to before the law (*P*<0.001). Adding a linear time trend as a predictor, the decrease was by 20.9 percent (*P*<0.001).There was a reduction in the monthly rate of traffic injuries related to alcohol by 10.5 percent as compared to before the law (*P*<0.001). Adding a linear time trend as a predictor, the decrease was by 24.8 percent (*P*<0.001).

**Conclusions:** Positive results followed from this new ‘zero-tolerance’ law implemented in 2012 in Chile. Chile experienced a significant reduction in alcohol-related traffic fatalities and injuries, being a successful public health intervention.

## Introduction

It is properly described the interrelation between alcohol drinking and road traffic crashes, the same way that are the main psychopharmacological consequences of alcohol on human conduct. Alcohol augments risk-taking actions and the chance of crashes by diminishing motor coordination and decision making in drivers^1^. Different blood alcohol concentrations (BAC) cause several neuro-motor changes: 0.03 g/dl, which corresponds to one serving of alcoholic beverage with 14 g of alcohol, causes loss of attention, false perception of speed, euphoria and difficulty in discriminating lighting conditions in space. Concentrations of 0.06 g/dl cause an increase in time of reaction and sleepiness and of 0.08 g/dl may lead to loss of peripheral vision, decrease in discerning lighting conditions and worse performance of routine activities^2, 3^.

Indirect examination of BAC by breathalyzers is a valuable method of producing precise BAC estimates during field impairment testing. BAC assessment is deemed a suitable method for deducing the influence of alcohol on driver´s performance. The relative risk of a crash starts to augment at 0.04-0.05 g/dl BAC, and rises steadily at BAC greater than 0.10, which is in line with international guidances advising that countries should embrace BAC limits of 0.05 or less ^1, 4-6^.

However, there is still disagreement as to what constitutes a dangerously high BAC, e.g.0.02 g/dl in Sweden, 0.03 g/dl in Japan, 0.05 g/dl in Germany and 0.08 g/dl in the United States. Even within the United States, illegal BAC limits have varied from state to state and have changed over the years ^7, 8^.

In the 2008 Global Status Report on Road Safety, the WHO clearly recommends institution of BAC limits of <0.05 g/dl in all drivers, with a stricter <0.02 g/dl limit for young/novice drivers. Despite the fact that 33% of WHO member states in the Americas have laws that meet or exceed the WHO recommendations, alcohol-associated crash rates remain extremely high in Central/South America and Mexico ^9^.

In 1990, the mortality in Chile due to traffic crashes was more than three times than in USA, Australia and the UK. Between 2001 and 2009, 8.1% of all traffic crashes, 10.2% of road injuries and 20.6% of road fatalities were associated to alcohol ^10^. In Chile, in 1994, there were 1679 deaths due to traffic crashes, with a rate of 19.6 per 100.000 inhabitants. Gender specific risks were 19.62 and 4.48 for men and women, respectively ^10^.

The University of California analyzed the severity of automotive injuries associated with BAC in increments of 0.01% in all US counties. The severity of life-threatening vehicle crashes increases significantly at BAC far lower than the US limit of 0.08%. They suggested that it might be appropriate to recalibrate the illegal US BAC limit closer to those used in Sweden and Japan ^11, 12^.

In the USA, a meta-analysis indicated that lowering the BAC limit from 0.10 to 0.08 g/dl resulted in a 14.8% reduction in alcohol-related traffic fatalities, and that lowering it to 0.05 would possibly produce an additional reduction of 6-18%^13, 14^. Similarly, a traffic legislation change carried out in Japan in 2002, which lowered the permissible BAC from 0.05 to 0.03, showed significant reductions in all traffic injuries^15^. The majority of high BAC drivers on US roads show no clinical signs of alcohol use disorder, but they were heavy drinkers^16-18^.

In Brazil, a new law introduced in 2008 lowered the BAC limit from 0.06 to 0.02. This was responsible for significant reductions in traffic injury and fatality rates, with a stronger effect for fatalities ^1, 19^.

Dos Santos Modelli et al. conducted a study to assess the association between high BAC and fatal victims of traffic crashes in the Brazilian Federal District. BAC higher than 0.6 g/L were detected in 44.2% of collision victims, and in 57.7% of victims of overturns ^20^.

With an intensification of street controls and a TV campaign to sensitize drivers about the alcohol risks while driving, the Law 20.580, popularly known as Zero Tolerance, was published in the Official Diary of Chile and became effective on March 15^th^, 2012 in the whole country of Chile.

The three main effects of the new law were 1) Reduce the legal limit of alcohol in blood while driving. 2) Implementation of a new test for measuring the level of alcohol, the evidential alcohol test, that can substitute the blood alcohol test speeding up the process. 3) Increase the penalties for violating the law.

The new law decreased the legal blood limit of alcohol for driving while impaired from 1 to 0.8 g/l and the legal blood alcohol limit for driving under the influence of alcohol from 0.5 to 0.3 g/l. The law is called Zero Tolerance because it doesn´t allow to drink any alcohol and operate a vehicle.

The previous procedure included the breath test and a blood test, for situations where the breath test indicates that is driving under the influence of alcohol. With the new law, this is maintained, but it also adds the possibility of substituting the blood test by an equivalent breath test, called the evidential breath test. Both tests, the blood alcohol test and the evidential breath test will have the same value as evidence. This new test is more effective, gives the result in 2 minutes and does not need to transport the driver to a health facility. This would increase the number of controls, helping to reduce demand at the health facilities. The alcohol controls were increased by Carabineros and by the National Service of Prevention and Rehabilitation for consumers of drugs and alcohol (Senda). At the same time, there were ambulances for the first time in the control locations.

The new law does not affect prison terms but is focused on increasing the length of license suspension. This new law is not retroactive. The new sanctions include:

1. Driving while impaired (>0.8 g/l) and first offense: 2 years suspension (previously it was from 6 to 12 months).
2. Driving while impaired and second offense: 5 years suspension.
3. Driving while impaired and third offense: license cancellation.
4. Driving while impaired and caused severe injuries or death: permanent revocation.

In case 4, only after 12 years it is possible to apply for reinstatement of license.

To my knowledge, this represents of the first formal investigations on the effectiveness of the new traffic law lowering the legal BAC limits in Chile with the goal of diminishing overall road traffic fatalities and injuries and associated to alcohol. It may inform similar countries where scientific data on this issue have not advanced.

## Methods

A retrospective review of national databases in Chile was conducted from January 2003 until December 2014 to evaluate total traffic mortality and injuries and due to alcohol before and after the enactment of the new road traffic law in March 2012. Monthly rates of traffic injuries and fatalities were measured per 100,000 inhabitants. The data sources included the National Institute of Statistics (INE) of Chile, the Ministry of Transport and Telecommunications of Chile (Conaset) and police reports (Carabineros de Chile), that are available in the public domain.

The definition taken from the data registries for “deaths related to alcohol” was the number of deaths found in traffic crashes where it was evidenced a driver driving while impaired or driving under the influence of alcohol as cause of the crash. The definition for “injuries related to alcohol” was the total number of injures in crashes including severe, moderate and mild injured where it was evidenced a driver driving while impaired or driving under the influence of alcohol as cause of the crash.

The study is not human subject research and did not require review by the Stanford University IRB, as determined by the Stanford University HRPP. IBM® SPSS® Statistics version 24.0 was used for the statistical analysis.

### Rationale for using Poisson regression

Generalized linear models type of Poisson regression analysis was used to analyze the impact of the law on the monthly incidence rates of events during the two time periods. The main interest lies in associating the incidence rate of events (deaths, injuries, deaths related to alcohol, and injuries related to alcohol) with the intervention. A second interest lies in associating the incidence rate of events with the different months of the year. As the number of occurrences of events is counted over a specific period, a Poisson distribution may be a reasonable way of modeling the distribution of the rates ^21^.

Because the mean number of occurrences must be positive, a natural way of modeling the conditional expectation of the response given the predictors month and intervention, is taking the exponential of the linear form of the equation. If we take the natural logarithm of each side of this equation, the natural logarithm will be the link function for a Poisson regression ^22^.

### Power analysis

Prior to collecting the post-law data, a power analysis was performed to calculate an appropriate sample size that would be sufficient to have adequate power.

The power calculation considered the Population at risk (76% of 17,248,450 = 13,108,822 population in 2011), event rate in the pre period (272 deaths in 2011 due to alcohol), years in the pre period and years in the post period, and gap (number of months between pre and post law). The power was calculated for a given alternative rate (i.e. something lower than 272/13,108,822), for the endpoint deaths due to alcohol.

The calculation was based on simulation with a regression discontinuity, assuming monthly or weekly data, with a (linear) trend, a possible yearly seasonal trend effect as well as a change at the time of the change being modeled.

The number of deaths in 2011 per 100,000 population was 2.07. If the law reduces that rate to 1.6 per 100,000 PY, the power would be 87%. These calculations assume 10 years of data prior to the law and 2 years after. The data from 2003 to 2014 would allow detecting a large effect of the law.

### Descriptive analysis

A descriptive analysis of the data was performed for the period from January 1^st^ 2003 to December 31^st^ 2014. The dependent variables were number of deaths, injuries, injuries related to alcohol, deaths related to alcohol, total crashes and total crashes related to alcohol.

Measures of central tendency and variability (mean, standard deviation, median, minimum and maximum) for each of these variables were analyzed ^23^.

Frequency analysis was used to describe categorical data divided into groups of before and after the law, calculating the number of observations per group, the percent of the total, the valid percent and the cumulative percent. There was complete data for all observations.

### Poisson regression

A series of additive Poisson log-linear models were used to evaluate the incidence rate of the number of occurrences of the event of interest per month from 2003 to 2014 as a function of the independent variables month and period.

The population in the databases was estimated at the beginning of each year. Linear interpolation from January to January was used to get a population number for each month. A new variable PY was created which is the number of person-years contributed during each month.

Another variable was the computed natural logarithm of PY (lnPY). This was used as an offset variable. The offset represents the log of some measure of exposure, in this case the population per month and is included with a regression coefficient constrained to 1.

A dichotomous variable was created called Period which was 1 in or after March 2012, and zero before February 2012. The Period category before the law was the reference subcategory.

The predictors included also the variable month category with its 12 months subcategories, taking January as the reference subcategory. It could be considered as a seasonal influence or annual predictable cyclical behavior of the series.

The Poisson regression analysis was designed using two different models, A and B. The difference was that in Model B a linear time trend (Yrs.Lin) continuous variable was added as a covariate. It was introduced to refer to the obvious tendency of the rates to change over time, being the simplest model of the time trends. The variable is calculated as the difference in years between each register date and the moment of law implementation in March 2012. The effects of the predictors in both models were combined as main effects.

The addition of a linear time trend was tested in a preliminary analysis of the data collected up to the year 2012. This showed that the effect of Period in model A is a decrease of 13.92 percent in the rate of deaths after the law as compared to before the law (*P*<0.001). The residual deviance for this model was 204.57 for 104 residual degrees of freedom (df).

When fitting a linear trend to the model, the effect of period in model B is a decrease of 4.3 percent in the rate after the law, and that is not significant (*P*=0.2108). The residual deviance in this model was 160.29 for 103 residual df.

The Chi-square statistic for the individual parameters was the Wald test. The confidence interval level type of Wald test was set at 95 percent. B and exponentiated B coefficients were obtained. The goodness of fit statistics were the deviance, scaled deviance, Pearson chi-square, scaled Pearson chi-square, log likelihood, AIC, finite sample corrected AIC, BIC and consistent AIC. The dispersion parameter for Poisson family was taken to be 1 for all models.

## Results

### Descriptive analysis

During the period from January 2003 to December 2014, there was a mean of 136.23 ± 17.078 deaths per month and a mean of 22.13 ± 8.608 deaths related to alcohol. There was a mean of 4347.30 ± 466.799 injuries per month and a mean of 417.37 ± 74.011 injuries related to alcohol. The mean number of crashes was 4746.03 ± 924.654 per month and the mean number of crashes related to alcohol was 369.10 ± 86.158 (Table 1).

**Table 1:** Measures of central tendency and variation for the response variables

**Variables**	**n**	**Mean**	**SD**	**Median**	**Min**	**Max**
Deaths	144	136.23	17.08	136.00	91	184
Injuries	144	4347.30	466.80	4327.00	3514	5706
Injuries alcohol	144	417.37	74.01	416.50	265	598
Deaths alcohol	144	22.13	8.61	21.00	8	48
Total crashes	144	4746.03	924.65	4697.50	3173	7401
Total crashes related to alcohol	144	369.10	86.16	375.00	209	546

Among the frequencies for the nominal-level variables, there were in total 144 months, as the sample size (N). In the first group before law implementation, there were 110 observations and in the other group 34, corresponding to a 76.4 percent of the total and valid percent and a 23.6 percent of the total and valid percent, respectively. The cumulative percent for both groups was 76.4 and 100.0, respectively.

**Figure 1:**
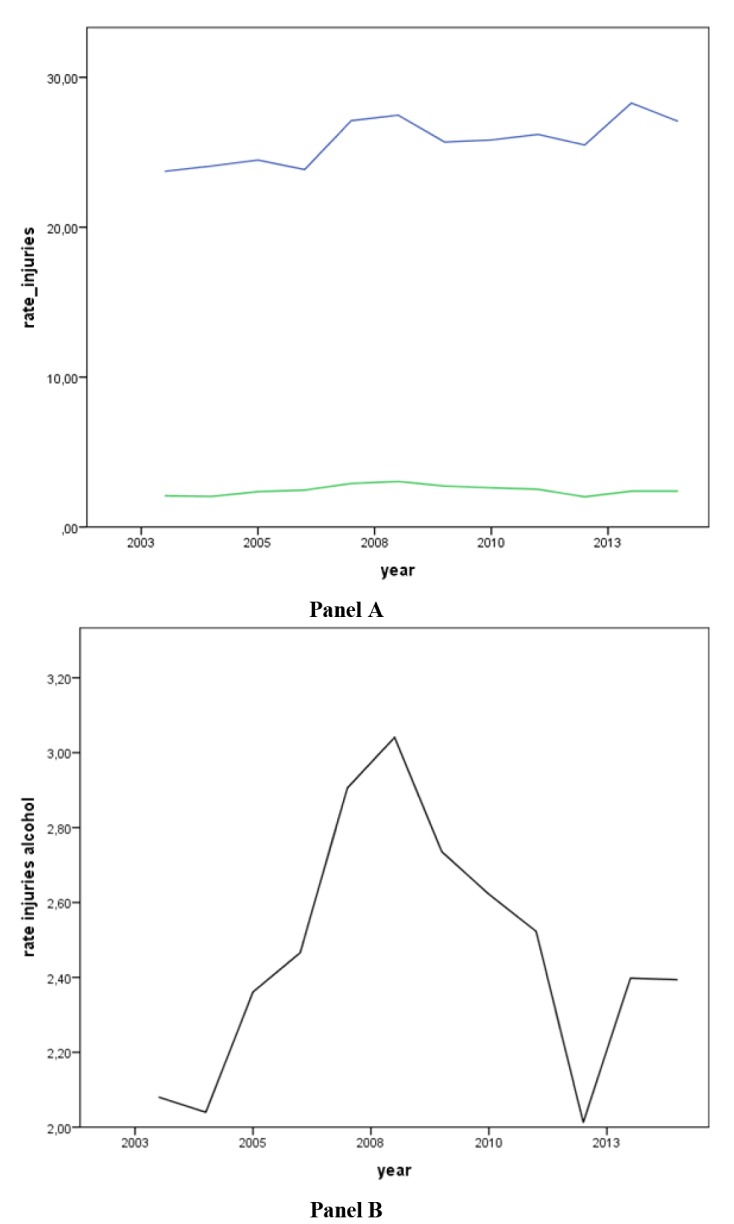
**Panel A.** Monthly trends in traffic injury rates in total (blue) and alcohol-related (green) per 100,000 inhabitants in Chile from January 2003 to December 2014. **Panel B.** Monthly trends in alcohol-related traffic injury rates per 100,000 inhabitants in Chile from January 2003 to December 2014.

**Figure 2:**
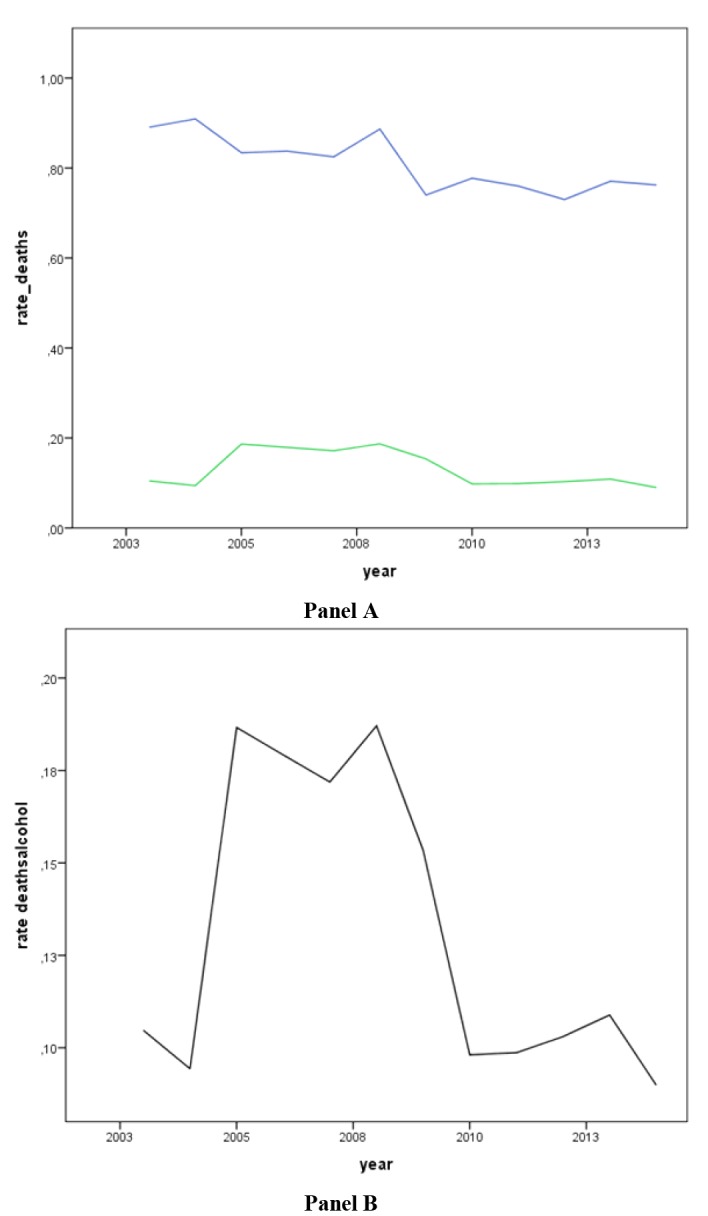
**Panel A.** Monthly trends in traffic fatality rates in total (blue) and alcohol-related (green) per 100,000 inhabitants in Chile from January 2003 to December 2014. **Panel B.** Monthly trends in alcohol-related traffic fatality rates per 100,000 inhabitants in Chile from January 2003 to December 2014.

The figures show the graph representation of the incidence rates of total deaths and injuries and related to alcohol per 100,000 population from January 2003 to December 2014 (Figure 1 and 2).

### Results of the multiple Poisson regression

### Model 1A. Deaths as the dependent variable

The Period variable showed a Exp (B) coefficient of 0.921 and this was statistically significant (*P*<0.001). This can be interpreted as the introduction of the law decreased the expected rate by a factor of 0.921 or by 7.9 percent, holding all other variables constant.

Among the month subcategories, only the Exp (B) for October and November (0.857 and 0.837, respectively) were statistically significant (*P*<0.001). This indicates that during these months there was a decrease in the expected rate of events by a factor of 0.857 and 0.837 or by 14.3 and 16.3 percent respectively, holding all other variables constant. These are differential effects in comparison with the January reference category.

The Deviance was 2.050, the Pearson Chi-Square was 2.041, the Scaled Deviance was 268.609 for 131 df, the Scaled Pearson Chi-Square was 267.417 for 131 df, the log likelihood was -619.978, AIC was 1265.956, finite sample corrected AIC was 1268.756, BIC was 1304.563 and consistent AIC was 1317.563.

### Model 1.B. Adding a linear time trend to Deaths

After the law the rate increased by a factor of 1.052 or by 5.2 percent, and this was statistically significant (*P*=0.045).

The continuous Yrs.Lin variable showed a Exp (B) of 0.978, meaning that the rate decreased over time by a factor of 0.978 or by 2.2 percent for a unit increase in Yrs.Lin, holding all other variables constant (*P*<0.001).

There were also statistically significant differences in the rates between the months of October and November (Exp(B)= 0.848 and 0.828 respectively, *P*<0.001) as compared to January.

The Deviance was 1.665, the Pearson Chi-Square was 1.669, the Scaled Deviance was 216.387 for 130 df, the Scaled Pearson Chi-Square was 216.920 for 130 df, the log likelihood was -593.867, AIC was 1215.734, finite sample corrected AIC was 1218.990, BIC was 1257.311 and consistent AIC was 1271.311.

### Model 2.A. Injuries as the dependent variable

There was a statistically significant Exp (B) for Period (1.059), meaning that the expected rate increased after the law by a factor of 1.059 or by 5.9 percent (*P*<0.001).

The 11 months of the year had statistically significant Exp(B) coefficients (*P*<0.001). Each of them showed a decrease in the expected rate as a differential effect with January.

The Deviance was 21.953, the Pearson Chi-Square was 21.954, the Scaled Deviance was 2875.779 for 131 df, the Scaled Pearson Chi-Square was 2876.006 for 131 df, the log likelihood was -2172.976, AIC was 4371.952, finite sample corrected AIC was 4374.752, BIC was 4410.559 and consistent AIC was 4423.559.

### Model 2.B. Adding a linear time trend to Injuries

There was a statistically significant (*P*<0.001) difference for Period (Exp (B) coefficient of 0.973), indicating that after the law there was a decrease instead in the rate by a factor of 0.973 or by 2.7 percent.

The variable Yrs.Lin showed a statistically significant (*P*<0.001) Exp (B) coefficient (1.014). This means that over time the number of injuries per month increased by a factor of 1.014 or by 1.4 percent for a unit increase in Yrs.Lin. Each of the 11 months were statistically significant (*P*<0.001), showing a decrease in the rate as a differential effect with January.

The Deviance was 16.875, the Pearson Chi-Square was 16.955, the Scaled Deviance was 2193.769 for 130 df, the Scaled Pearson Chi-Square was 2204.182 for 130 df, the log likelihood was -1831.971, AIC was 3691.942, finite sample corrected AIC was 3695.198, BIC was 3733.520 and consistent AIC was 3747.520.

### Model 3.A. Alcohol-related Deaths

There was a statistically significant (*P*<0.001) difference for period, with a reduction after the law in the number of deaths related to alcohol by a factor of 0.719 or by 28.1 percent as compared to before the law.

The months of May, September and November were statistically significant. It was seen an increase in the rate during May and September and a decrease in November as a differential effect with January (Table 2).

**Table 2:** Generalized Linear Models analysis for a Poisson Probability Distribution with Log Link Function, showing the Parameter Estimates for Deaths related to alcohol

**Parameter**	**B**	**Hypothesis test**			**Exp(B)**	**Exp(B) 95% CI**	
		**Wald Chi-Square**	**df**	**P value**		**Lower**	**Upper**
Intercept	-13.544	45818.079	1	0.001	1.311E-6	1.158E-6	1.484E-6
Month=12	0.150	3.020	1	0.082	1.162	0.981	1.376
Month=11	-0.234	6.031	1	0.014	0.791	0.657	0.954
Month=10	-0.104	1.281	1	0.258	0.901	0.752	1.079
Month=9	0.200	5.487	1	0.019	1.221	1.033	1.444
Month=8	0.024	0.071	1	0.789	1.024	0.860	1.219
Month=7	0.005	0.003	1	0.958	1.005	0.843	1.197
Month=6	0.152	3.080	1	0.079	1.164	0.982	1.378
Month=5	0.233	7.531	1	0.006	1.262	1.069	1.490
Month=4	0.153	3.149	1	0.076	1.166	0.984	1.381
Month=3	0.082	0.869	1	0.351	1.085	0.914	1.289
Month=2	0.079	0.822	1	0.365	1.082	0.912	1.285
Month=1.	0.000^a^	.	.	.	1.000	.	.
Period=1	-0.330	53.496	1	0.001	0.719	0.658	0.785
Period=0	0.000^a^	.	.	.	1.000	.	.
Scale	1.000^b^						

Dependent Variable: deaths, alcohol Model: (Intercept), month, period, offset = lnPY ^a^ Set to zero because this parameter is redundant ^b^ Fixed at the displayed value

The Deviance was 2.965, the Pearson Chi-Square was 2.916, the Scaled Deviance was 388.458 for 131 df, the Scaled Pearson Chi-Square was 382.003 for 131 df, the log likelihood was -544.639, AIC was 1115.278, finite sample corrected AIC was 1118.078, BIC was 1153.886 and consistent AIC was 1166.886.

### Model 3.B. Adding a linear time trend to alcohol-related Deaths

There was a statistically significant (*P*<0.001) difference for Period, with a decrease after the law in the number of deaths related to alcohol by a factor of 0.791 or by 20.9 percent, as compared to before the law.

The variable Yrs.Lin showed a statistically significant Exp (B) coefficient (*P*=0.031), meaning that for a unit increase in time there was a decrease in the rate by a factor of 0.984 or by 1.6 percent.

May, September and November were statistically significant. It was seen an increase in the rate during the months of May and September, and a decrease in November as a differential effect with January (Table 3).

**Table 3:** Generalized Linear Models analysis for a Poisson Probability Distribution with Log Link Function, showing the Parameter Estimates for alcohol-related Deaths adding a linear time trend

**Parameter**	**B**	**Hypothesis test**			**Exp(B)**	**Exp(B) 95% CI**	
		**Wald Chi-Square**	**df**	**P value**		**Lower**	**Upper**
Intercept	-13.616	36090.431	1	0.001	1.221E-6	1.061E-6	1.405E-6
Month=12	0.142	2.720	1	0.099	1.153	0.974	1.366
Month=11	-0.242	6.419	1	0.011	0.785	0.652	0.947
Month=10	-0.112	1.473	1	0.225	0.894	0.747	1.071
Month=9	0.192	5.066	1	0.024	1.212	1.025	1.433
Month=8	0.016	0.033	1	0.857	1.016	0.853	1.210
Month=7	-0.003	0.001	1	0.973	0.997	0.836	1.188
Month=6	0.144	2.767	1	0.096	1.155	0.975	1.368
Month=5	0.225	7.021	1	0.008	1.252	1.060	1.479
Month=4	0.145	2.829	1	0.093	1.157	0.976	1.370
Month=3	0.074	0.708	1	0.400	1.077	0.906	1.279
Month=2	0.079	0.823	1	0.364	1.082	0.912	1.285
Month=1.	0.000^a^	.	.	.	1.000	.	.
Period=1	-0.235	13.669	1	0.001	0.791	0.698	0.896
Period=0	0.000^a^	.	.	.	1.000	.	.
Yrs.Lin	-0.016	4.637	1	0.031	0.984	0.970	0.999
Scale	1.000^b^						

Dependent Variable: deaths, alcohol Model: (Intercept), month, period, offset = lnPY ^a^ Set to zero because this parameter is redundant ^b^ Fixed at the displayed value

The Deviance was 2.952, the Pearson Chi-Square was 2.902, the Scaled Deviance was 383.821 for 130 df, the Scaled Pearson Chi-Square was 377.277 for 130 df, the log likelihood was -542.320, AIC was 1112.641, finite sample corrected AIC was 1115.897, BIC was 1154.218 and consistent AIC was 1168.218.

### Model 4.A. Alcohol-related Injuries

There was a statistically significant (*P*<0.001) difference for period, with a reduction in the rate after the law by a factor of 0.895 or by 10.5 percent.

February, March, April, June, July, August, October and November were statistically significant (*P*<0.001). It was observed a decrease in the rate during each of these months as a differential effect with January.

The Deviance was 10.876, the Pearson Chi-Square was 10.778, the Scaled Deviance was 1424.737 for 131 df, the Scaled Pearson Chi-Square was 1411.930 for 131 df, the log likelihood was -1278.017, AIC was 2582.034, finite sample corrected AIC was 2584.834, BIC was 2620.641 and consistent AIC was 2633.641.

### Model 4.B. Adding a linear time trend to Alcohol-related Injuries

There was a statistically significant (*P*<0.001) difference for Period, with a decrease in the rate after the law by a factor of 0.752 or by 24.8 percent.

The Yrs.Lin variable had a statistically significant (*P*<0.001) Exp (B) coefficient (1.030), meaning that the rate increased by a factor of 1.030 or by 3 percent for each unit increase in time.

February, March, April, June, July, August, October and November were statistically significant (*P*<0.001). It was observed a decrease in the rate during each of these months as a differential effect with January.

The Deviance was 8.679, the Pearson Chi-Square was 8.655, the Scaled Deviance was 1128.326 for 130 df , the Scaled Pearson Chi-Square was 1125.193 for 130 df, the log likelihood was -1129.811, AIC was 2287.622, finite sample corrected AIC was 2290.878, BIC was 2329.200 and consistent AIC was 2343.200.

## Discussion

This study used a descriptive analysis and a series of additive Poisson Log-linear Models to demonstrate the effect of the new traffic law in Chile, accounting for the month influence, a linear time trend and population exposure.

It is shown in the model for total deaths that after the law there was a statistically significant decrease in the rate by 7.9 percent. In the same model, there was a statistically significant reduction by 14 percent during October and by 16.3 percent during November, as compared to January.

However, after adding a linear time trend to this model, the rate after the law showed instead a statistically significant increase by 5.2 percent. Similar changes occurred during the months of October and November, with a decrease by 15.2 and 17.2 percent, respectively. This model with and without a linear time trend had acceptable Goodness of Fit values.

In the model evaluating total injuries, it was observed a statistically significant increase in the rate by 5.9 percent after the law. This contrasts with the results obtained after adding a linear time trend, where the rate after the law showed a statistically significant decrease by 2.7 percent instead, even though the rate increased over time.

In the model evaluating deaths related to alcohol, it was observed a statistically significant decrease after the law by 28.1 percent. It was observed a statistically significant increase in the rate during May and September and a decrease during November, as compared to January.

When adding a linear time trend as a predictor, the rate showed again a statistically significant decrease, by 20.9 percent. The months of May and September showed again a statistically significant increase in the rate and November a decrease as compared to January. The Goodness of fit analysis of this model with and without including the time covariate indicated an adequate fit.

The model evaluating injuries related to alcohol showed a statistically significant decrease in the rate by 10.5 percent after the law. When adding a linear time trend to this model, there was a bigger drop in the rate, by 24.8 percent instead, even though the rate increased over time.

It is interesting to note that, after the law, the rate of traffic fatalities related to alcohol, in the model adjusting for time, decreased by 20.9 percent. However, the rate of total traffic fatalities, adjusting for the time variable as well, increased after the law by 5.2 percent.

There was an increase in the rate of total injuries after the law by 5.9 percent. Nonetheless, the rate of injuries related to alcohol decreased by 10.5 percent. When adding a linear time trend to the model, the rate of total injuries decreased by 2.7 percent after the law even though the rate increased over time. This drop was greater for the rate of injuries related to alcohol in the model adding a linear trend, with a decrease of 24.8 percent even though the rate increased over time.

This supports that the primary effect of the new traffic law was to change the behavior of people who intended to drive a motor vehicle after drinking, which affected significantly alcohol associated fatalities and injuries but had a minor effect in the overall traffic fatalities and injuries.

The introduction of a legal BAC limit has been effective in the UK, Canada, The Netherlands, and Japan ^7^. This study shows similar results regarding events related to alcohol in comparison to studies from other countries. In the USA, the introduction of the 0.08% BAC Law is controversial, but appears to have reduced alcohol-impaired driving ^24^.

However, a methodological issue should be considered in the interpretation of the results of the model for total injuries. The Goodness of fit statistics did not indicate an adequate fit of this model. This may be due to missing covariates, overdispersion, or the need for a negative binomial regression.

The problem of overdispersion can be caused by the specification of the model. For instance, important predictor variables may have been left out. Other explanatory variables were not considered as it was not possible to systematically control for external confounders such as other new traffic laws, campaigns, and overall alcohol consumption.

One of the reasons for overdisperison in Poisson regression is heterogeneity among sample members. Corrective measures include using the deviance or Pearson chi-Square divided by the df as an estimate of the dispersion parameter. Afterwards the estimated standard errors and the Wald chi-Square statistics are different, though the parameter estimates will be identical to the original fit.

Other possibilities would be to consider different methods, for example to model individual crashes instead of dealing with aggregate data. This was not possible because the Legal Medicine reports in Chile and hospital medical records were not available due to Data Protection and Confidentiality Policies. The study was designed collecting data at an aggregate level from datasets available in the public domain.

The same concern about overdispersion applies to the model of injuries related to alcohol. It seems the specification of the injuries models may need other predictor variables to be included or that different methods should be considered, for example to model individual crashes instead of dealing with aggregate data. However, the precision of a model does not guarantee to reflect the reality.

## Conclusions

The new road traffic law implemented in Chile in March 2012 had a significant effect on declining traffic injuries and fatalities associated with alcohol, suggesting positive results from this new law enactment. The impression is that there was no large effect in overall traffic fatalities and injuries after the introduction of the law. On balance, the Chilean policy appears to have been a successful public health measure.

The findings of this study may be helpful for other countries coping with the problems of drunk driving. Additionally, this study provides the chance to develop further a public health approach and future research hypotheses for the drink and driving problem in Chile.

## Conflict of interest statement

There is no conflict of interests.

## Funding

No grants were involved in supporting this work.

## Highlights

- The law had a statistically significant effect on reducing alcohol-related traffic injuries and fatalities.
- There was no large effect in overall traffic fatalities and injuries after the law .
- On balance, the Chilean policy appears to have been a successful public health measure.
- This study may be helpful for other countries coping with drunk driving problems.
